# Supplementary material for: Outcomes of Temporary Hemiepiphyseal Stapling for Correcting Genu Valgum in Children with Multiple Osteochondromas: A Single Institution Study
Source: Children (Basel). 2021 Apr 8;8(4):287. doi: 10.3390/children8040287 (PMC8068200; doi:10.3390/children8040287)
Supplement: Supplementary file 1 [file children-08-00287-s001.pdf]

INFERIOR OUTCOMES OF TEMPORARY HEMIEPIPHYSEAL STAPLING FOR CORRECTING GENU VALGUM IN CHILDREN WITH MULTIPLE OSTEOCHONDROMAS. A SINGLE INSTITUTION STUDY

Table S1. List of radiographic parameters

Parameter	Description	Normal range
Femoral length (FL)	Line from the top center of the femoral head to a line parallel to the distal medial and lateral condyles	
Tibial length (TL)	Line from the center of the intercondylar eminence to the center of the tibial plafond (midpoint between the medial and lateral malleoli at distal tibia)	
Fibular length (FbL)	Line from the apex to the lowest end of the fibula	
Mechanical axis of the lower extremity	Line from the center of the femoral head to the center of the tibial plafond	
Absolute Mechanical axis deviation (MAD mm)	The perpendicular distance from the mechanical axis of the lower extremity line to the center of the knee joint, expressed in mm	8 ± 7 mm medial. Genu valgus deformity was identified based on increased lateral deviation of the MAD
Relative Mechanical axis deviation (MAD %)	The perpendicular distance from the mechanical axis of the lower extremity line to the center of the knee joint, expressed as a percentage to one half of the width of the tibial plateau (to eliminate the bias created by the different tibial width in each patient)	
Femoral mechanical axis	Line between the center of the femoral head and the midpoint between the medial and lateral condyles	
Femoral anatomic axis	The mid-diaphyseal line of the femur	
Tibial mechanical axis	Line from the center of the knee joint to the center of the ankle plafond	
Tibial anatomic axis	The mid-diaphyseal line of the tibia. It usually corresponds to the tibial mechanical axis	
Mechanical hip–knee–ankle (HKA) axis	Angle between a line drawn from the center of the femoral head to the center of the knee joint and a line drawn from the center of the knee joint to the center of the tibial plafond. Positive values for varus knee, negative values for valgus knee	1° ± 2° varus
Mechanical lateral distal femoral angle (mLDFA)	The lateral angle formed between the lines of the mechanical femoral axis and the articular surface of the distal femur	88° ± 2°
Anatomical lateral distal femoral angle (aLDFA)	The medial angle formed between the line of the mechanical tibial axis and a line tangent to the joint surface of the proximal tibial plateau	81° ± 2°
Mechanical medial proximal tibial angle (mMPTA)	The medial angle formed between the line of the mechanical tibial axis and a line tangent to the joint surface of the proximal tibial plateau	87° ± 2°
Anatomical medial proximal tibial angle (aMPTA)	The medial angle formed between the line of the anatomical tibial axis and a line tangent to the joint surface of the proximal tibial plateau	87° ± 2° It normally corresponds to the mMPTA.

Table S2. Radiographic parameters: the inter-observer reliability

**Radiographic parameters**	**ICC* (95% IC)**
Femur length (mm)	1.00 (0.99 – 1.00)
Tibia length (mm)	0.99 (0.98 – 1.00)
Fibula length (mm)	1.00 (0.99 – 1.00)
HKA (°)	0.94 (0.90 – 0.96)
MAD (mm)	0.99 (0.98 – 1.00)
MAD (%)	0.99 (0.98 – 1.00)
aLDFA (°)	0.86 (0.77 – 0.91)
mLDFA (°)	0.86 (0.73 – 0.94)
MPTA (°)	0.89 (0.82 – 0.93)
* ICC: Interclass Correlation	

Table S3. Modified Clavien-Dindo-Sink Complication Classification System [27-28]

Grade	Definition	Examples
**I**	A complication that does not result in deviation from routine follow-up in the postoperative period and has minimal clinical relevance and requires minimal treatment (e.g., antiemetics, antipyretics, analgesics, diuretics, electrolytes, antibiotics, and physiotherapy) or no treatment	Fever (>37.5°) or nausea, present after surgery, but quickly resolved spontaneously or after planned pharmacological treatment
**II**	A deviation from the normal postoperative course (including unplanned clinic/office visits) that requires outpatient treatment, either pharmacological or close monitoring as an outpatient	The child needed unplanned additional drugs, physiotherapy, or exams in case of pain, neurapraxia or thrombosis
**III**	A complication that is treatable but requires surgical, endoscopic, or interventional radiology procedure(s), or an unplanned hospital readmission	A hospital re-admission was necessary, when surgical reinterventions were needed like reoccurrence of osteochondromas previously excised, hypo- or over-correction, plate malposition or breakage of hardware, infection
**IVa**	A complication that is life or limb-threatening, and/or requires ICU* admission, a complication with potential for permanent disability but treatable, a complication that may require organ/joint resection/replacement. No long-term disability	
**IVb**	A complication that is life or limb-threatening, and/or requires ICU admission, a complication that is not treatable, a complication that requires organ/joint resection/replacement or salvage surgery. With long-term disability	
**V**	Death	Not observed in any case

* ICU: Intensive Care Unit

Table 4. Predictors of MAD (%) and Correction velocity. Multivariable linear regression models.

		MAD[a] post-operative (%)			Correction velocity		
**Predictors**		**Coef[b]**	**95% CI**	**P-value**	**Coef**	**95% CI**	**P-value**
Group	MO	10.85	-1.45, 23.16	0.083	-0.73	-1.38, -0.08	**0.029**
Gender	Male	-3.89	-10.16, 2.37	0.222	0.40	0.00, 0.80	**0.048**
Age at stapling		0.89	-1.93, 3.70	0.535	0.01	-0.19, 0.20	0.960
Height (z-score)		4.11	1.59, 6.62	**0.002**	0.00	-0.23, 0.23	0.996
Weight (z-score)		-4.20	-9.11, 0.72	0.094	0.14	-0.22, 0.51	0.433
Affected side	Right	-4.20	-9.11, 0.72	0.094	-0.03	-0.20, 0.14	0.745
Site	Distal femur (ref.)	-	-	-	-	-	-
	Proximal tibia	2.64	-4.76, 10.05	0.482	-0.40	-0.90, 0.10	0.112
	Distal femur & proximal tibia	-4.28	-20.26, 11.69	0.597	0.79	-0.58, 2.15	0.258
	Other	0.37	-10.64, 11.37	0.948	-0.31	-0.80, 0.18	0.213
MAD pre-operative, %		0.41	0.23, 0.58	**<0.001**	0.02	0.00, 0.03	**0.022**
Bilaterality		-1.84	-18.77, 15.09	0.830	-0.10	-1.02, 0.81	0.828

[a] MAD: Mechanical Axis Deviation; [b] Coef: adjusted mean difference, for continuous variables coefficients were reported for 1-point increase on the respective unit of measurement

Table S5. Adjustment comparisons between MO *versus* idiopathic cohort on post-operative MAD and correction velocity. Linear regression models.

Comparisons between MO *versus* idiopathic cohort using a Propensity Score approach

To adjust the comparisons between MO *versus* idiopathic cohort on HeS outcomes, we performed different analyses using a Propensity Score (PS) approach. A PS for the likelihood to have been diagnosed with MO was calculated using a logistic regression model from the following covariates: gender, Age at stapling, height z-score, weight z-score, affected side, site, bilaterality, and pre-operative MAD. Linear regression models were estimated including as a predictor the diagnostic group variable along with the PS. Besides, the PS was applied to create more balanced samples with a 1:1 ratio between MO and idiopathic children, using the nearest neighbor matching without replacement, with and without a caliper distance of 0.40 standard deviation of the PS distribution.

The analyses results, along with the estimates deriving from multivariable analyses, were reported in Table S4.

	MAD[a] post-operative (%)			Correction velocity		
**Adjustment Method**	**Coef[b]**	**95% CI**	**P-value**	**Coef**	**95% CI**	**P-value**
Full covariate	10.66	-2.18, 22.29	0.107	-0.71	-1.37, -0.04	0.037
PS[c] as covariate	16.09	3.75, 28.42	0.011	-0.81	-1.63, 0.01	0.053
PS Matching, nearest neighbour †	14.37	2.41, 26.34	0.020	-0.78	-1.25, -0.31	0.002
PS Matching, nearest neighbour with caliper 0.40‡	15.96	-10.91, 42.83	0.215	-0.87	-1.66, -0.08	0.035

[a] MAD: Mechanical Axis Deviation; [b] Coef: adjusted mean difference, for continuous variables coefficients were reported for 1-point increase on the respective unit of measurement; [c] PS: Propensity Score

† N=62 (31 MO, 31 Idiopathic) for post-operative MAD analysis, N=54 (27 MO, 27 Idiopathic) for correction velocity analysis

‡ N=22 (11 MO, 11 Idiopathic) for post-operative MAD analysis, N=20 (10 MO, 10 Idiopathic) for correction velocity analysis
